# Evaluation of Tissue Ischemia/Reperfusion Injury in Lung Recipients Supported by Intraoperative Extracorporeal Membrane Oxygenation: A Single-Center Pilot Study

**DOI:** 10.3390/cells11223681

**Published:** 2022-11-19

**Authors:** Fiorella Calabrese, Federica Pezzuto, Francesco Fortarezza, Francesca Lunardi, Eleonora Faccioli, Giulia Lorenzoni, Annalisa Boscolo, Nicolò Sella, Dario Gregori, Marco Schiavon, Paolo Navalesi, Andrea Dell’Amore, Federico Rea

**Affiliations:** 1 Department of Cardiac, Thoracic, Vascular Sciences, and Public Health, University of Padova, 35128 Padova, Italy; 2 Institute of Anesthesia and Intensive Care, Padova University Hospital, 35128 Padova, Italy; 3 Department of Medicine, University of Padova, 35128 Padova, Italy

**Keywords:** ischemia/reperfusion injury, primary graft dysfunction, extracorporeal membrane oxygenation

## Abstract

Intraoperative veno-arterial (VA) extracorporeal membrane oxygenation (ECMO) as intraoperative hemodynamic support during lung transplantation is becoming a standard practice due to promising clinical results. Nevertheless, studies on tissue/molecular pathways investigating ischemia/reperfusion injury are still lacking. Patients receiving a bilateral lung transplantation between January 2012 and December 2018 at the University Hospital of Padova were included in this retrospective single-center observational study. The present study aimed to investigate ischemia/reperfusion injury in 51 tissue specimens obtained from 13 recipients supported by intraoperative VA-ECMO and 38 who were not. Several tissue analyses, including apoptosis evaluation and inducible nitric oxide synthase expression, were performed on the biopsies at the time of transplantation. Lung samples from the ECMO group (both pre- and post-reperfusion) were comparable, or for some parameters better, than samples from the non-ECMO group. Leukocyte margination was significantly lower in the ECMO group than in the non-ECMO group. Primary graft dysfunction, mainly at 24 and 48 h, was correlated with the tissue injury score of the post-reperfusion biopsy. The interquartile ranges for all morphological parameters showed high grade variability between pre- and post-reperfusion in the non-ECMO group. These preliminary data support the use of intraoperative ECMO based on lower lung tissue ischemia/reperfusion injury. Larger case series are mandatory to confirm our findings.

## 1. Introduction

Ischemia/reperfusion (I/R) injury is a major cause of primary graft dysfunction (PGD) following lung transplantation and is associated with significant morbidity and mortality [1]. I/R injury has been shown to depend on the inability to maintain and repair homeostatic barriers that promote pulmonary function, namely, the airway epithelium and the vascular endothelium [1]. The leading mechanism of this phenomenon may be found in the preservation strategies of organs selected for transplantation [2]. Although the period of cold ischemic storage is kept as short as possible (4 to 8 h) and several optimizations have been performed to improve the technique, a series of events, such as oxidative stress, sodium pump inactivation, intracellular calcium overload, iron release, and induction of cell death, may occur, with severe impacts on graft function [3]. A newly implanted graft is especially vulnerable during the reperfusion phase. Controlled reperfusion strategies are thought to be crucial for good graft outcomes [4]. Currently, a standard procedure in lung transplantation consists of a gradual opening of the clamp on the pulmonary artery in 5–10 min [5]. Although cardiopulmonary bypass (CPB) can control reperfusion for long periods, several complications (high intraoperative blood turnover, bleeding in the postoperative period, disseminate inflammatory response) limit its use [6]. Veno-arterial extracorporeal membrane oxygenation (VA-ECMO) was conceived as an alternative to CPB and is now increasingly used in several lung transplantation centers [6]. The benefit of VA-ECMO as intraoperative support during lung transplantation has been widely discussed in the literature, often with contradictory results [7,8,9,10]. However, recent advances in ECMO systems and management strategies have improved the clinical outcomes of bridges to lung transplantation over the last decade [11,12,13,14,15,16]. In addition, prolongation of VA-ECMO in a peripheral configuration after surgery has recently been shown to be useful in reducing the risk of PGD in patients at higher risk (such as those with pulmonary hypertension and cardiac failure) [11,12,13]. These findings have introduced the concept of ECMO as a tool for the prevention of ischemic reperfusion injury, from its routine intraoperative use in all transplanted patients to its extension to intensive care on the basis of pre-defined criteria well described by the Vienna group [11,12,13].

Studies on tissue/molecular pathways investigating I/R injury in patients treated with VA-ECMO are still lacking. Thus, the main goal of this research was to assess the differences in ischemic tissue damage between lung tissue specimens from patients who received intraoperative VA-ECMO support and specimens from non-ECMO support patients during lung transplantation.

## 2. Materials and Methods

### 2.1. Study Design and Population

Between January 2012 and December 2018, among the 138 patients who underwent lung transplantation at our institution, 49 (36%) patients were successfully transplanted with intraoperative VA-ECMO support (ECMO patients). The remaining 89 (64%) patients did not require intraoperative VA-ECMO support (non-ECMO patients).

In our center, intraoperative VA-ECMO support was achieved with central aorto-right atrium cannulation trough a clamshell incision. Briefly, the main indications for intraoperative VA-ECMO support were pulmonary artery hypertension, low cardiac index, and severe cardiomegaly with impaired right heart function, or clamping of the first pulmonary artery or single-lung ventilation causing cardiorespiratory instability. As previously described by other authors [11,12,13], our indications for the prolongation of ECMO in the intensive care unit (ICU) are: marginal donors, long ischemic time (>7 h), high-risk recipient (severe pulmonary artery hypertension with right ventricular impairment), severe pulmonary artery hypertension after reperfusion (>2/3 the systemic pressure), high requirement of inotropes, signs of early reperfusion lung injury with rapid worsening of respiratory and hemodynamic parameters, and necessity of aggressive ventilation to maintain acceptable pO_2_, pCO_2_, and pH values.

**Figure 1:**
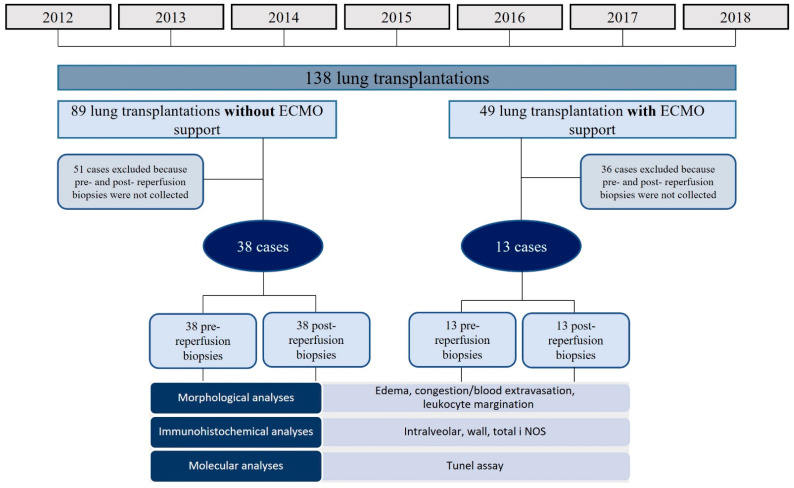
Flow chart describing the study population and design.

The present study population included 51 patients. The inclusion criteria were: (a) patients older than 18 years who underwent bilateral lung transplantation; (b) availability of pre- and post-reperfusion lung biopsy; (c) availability of pre-reperfusion (immediately before transplantation) and post-reperfusion (on the first implanted lung, 90 min after in vivo reperfusion of the first implanted lungs) biopsies (Figure 1).

The exclusion criteria were: (a) patients who underwent volumetric reductions in the graft before pulmonary reperfusion; (b) patients undergoing re-transplantation or multi-organ transplantation; (c) patients transplanted with organs removed with ex vivo perfusion methods (Organ Care System-OCS); (d) patients bridged with ECMO to lung transplantation.

Recipients were transplanted by three experienced surgeons following a standardized strategy for extracorporeal support. PGD was calculated according to the latest International Society for Heart and Lung Transplantation (ISHLT) recommendation 2 h after intensive care unit admission (t0), 24 h (t24), 48 h (t48), and 72 h (t72) after transplantation [17].

The research was carried out in accordance with the principles of the Helsinki Declaration of 1975, as revised in 1983, and the guidelines for Good Clinical Practice. The institutional ethics committee approved the study (4539/AO/18), and all patients gave informed consent.

### 2.2. Morphological Analyses, Terminal Deoxynucleotidyl Transferase dUTP Nick End Labeling (TUNEL) Assay, and Immunohistochemistry

Morphological analyses, TUNEL assays, and immunohistochemical tests were carried out on serial sections of all pre- and post-reperfusion lung samples collected from the middle lobe and lingula as previously described [18].

Morphological analyses included the evaluation of several parameters (edema, congestion/blood extravasation, and leukocyte margination), which were graded using a four-tier scoring system (score range: 0–3). Different morphological parameters (edema, blood extravasation, and leukocyte margination) were quantified and graded with the following scoring system: score 0 = absent; score 1 = mild, <30% of analyzed tissue; score 2 = moderate, 30–50% of analyzed tissue; and score 3 = severe, >50% of analyzed tissue. Two pathologists (F.C. and F.P.) independently quantified the histological parameters. Discordant cases were discussed, and a shared diagnosis was achieved.

The detection of apoptotic cell death was also investigated using TUNEL assays. Briefly, paraffin-embedded lung tissue sections were rehydrated and treated with proteinase K solution for permeation. The slides were immersed in terminal deoxynucleotidyl transferase (TdT) labeling buffer and covered with anti-bromodeoxyridine (anti-BrdU) and incubated with sterptavidin–horseradish peroxidase (HRP) solution. Diaminobenzidine (DAB) was used as the chromogen, and cells containing fragmented nuclear chromatin—the characteristic of apoptosis—exhibited brown nuclear staining. At least 300 cells were counted in 3 high-power fields. Apoptotic index (AI) was expressed as the number of TUNEL-positive cells/total cell number ×100. Quantitative evaluation was performed by computer-assisted morphometric analysis (Image Pro-Plus Version 5).

Immunohistochemistry was performed using the primary monoclonal antibody anti-inducible nitric oxide synthase (iNOS, clone SP126; Abcam, Cambridge, UK). At least 5 high-power fields were analyzed in each sample, and the mean positive cell number/mm^2^ of tissues was reported, distinguishing intra-alveolar (mainly macrophages) and wall (mainly epithelial cells and more rarely endothelial cells) compartments.

AI and iNOS were further scored based on the quartile values (1 < Q1, 2 between Q1 and Q3, 3 > Q3). A combined score system that included morphological score, AI, and alveolar iNOS was built. In each case, a total score was computed as the sum of the three-parameter scores, ranging from 0 to 15.

### 2.3. Real-Time PCR for iNOS Expression

The analysis was performed on a subset of 24 frozen samples to ascertain the occurrence of iNOS expression at the messenger RNA (mRNA) level. Lung fragments of 1 cm × 0.8 cm were stored in RNA-later (Qiagen, Hilden, Germany), kept at 4 °C until freezing into liquid nitrogen, and then preserved at −80 °C.

Total RNA was extracted from frozen lung tissues, and quantitative determination of mRNA levels of the iNOS gene was performed in triplicate on a Light Cycler 480 II (Roche Applied Science, Mannheim, Germany) using SYBR green-based quantification. Glyceraldehyde-3-phosphate dehydrogenase (GAPDH) was used as an internal control to normalize target genes. The average cycle threshold (CT) was determined, and ΔCT was calculated by normalization with the housekeeping gene. Different expressions were then evaluated via the ΔΔCT method [18].

### 2.4. Statistical Analyses

Descriptive statistics are reported as I quartile/median/III quartile for continuous variables and percentages (absolute numbers) for categorical variables. Wilcoxon and chi-squared tests were performed to compare the distributions of continuous and categorical variables, respectively. To assess the effect of ECMO on morphological parameters (leukocyte margination, apoptotic index, and INOS) pre- and post-reperfusion, regression models were employed. Linear regression models were employed for continuous parameters, while logistic regression models were estimated for binary parameters. For each parameter, two models were estimated, one for each time-point (pre- and post-reperfusion), for ECMO vs. non-ECMO patients. The variables edema and congestion were dichotomized due to the poor representation of the categories (0: scores 0–1 and 1: score 2–3)

The variability in the parameters, pre- and post-reperfusion in ECMO and non-ECMO patients and between ECMO and non-ECMO patients, was evaluated in the interquartile range, considering 10,000 bootstrap repetitions. To assess the effects of the clinical parameters of interest, the effects of body mass index (BMI) and pulmonary hypertension on the variability of morphological parameters were estimated using generalized estimating equation models. The models included BMI and pulmonary hypertension as predictors for modeling the dispersion of morphological parameters. Survival distribution at follow-up was evaluated using the Kaplan–Meier method. Analyses were performed using R software, together with the packages rms, survival, survminer, and geepack. All the tests performed were two-sided; *p* <  0.05 was considered significant.

## 3. Results

The main clinical recipient and donor characteristics of the ECMO and non-ECMO groups are summarized in Table 1 and Table 2.

The clinical data showed that the patients who underwent ECMO support showed a higher preoperative risk profile than non-ECMO patients, with higher median values of pulmonary artery pression and higher BMIs (median value of 22 in ECMO patients vs. 19.50 in non-ECMO patients). When considered as confounding factors, they did not affect the histological evaluations.

**Figure 2:**
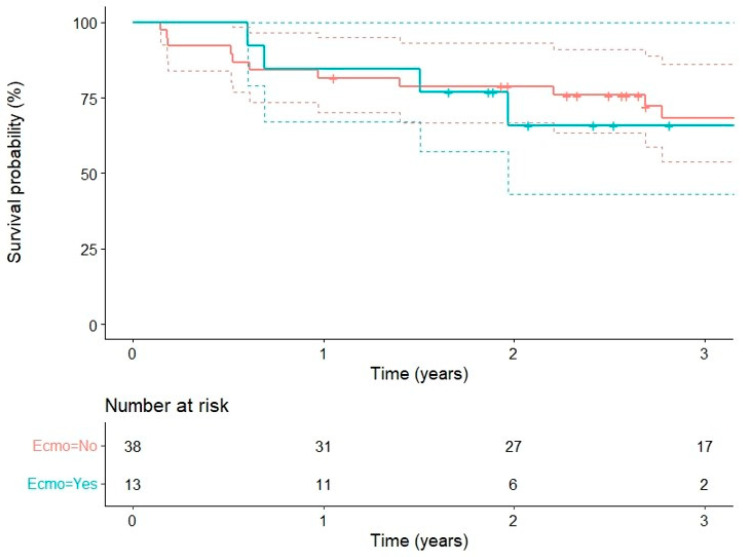
Kaplan–Meier survival curves (solid lines) and 95% confidence interval (dashed lines) for ECMO and non-ECMO patients.

Two patients in the non-ECMO group died early, while no patient in the ECMO group died within 30 days after lung transplantation. At 24 h post-transplantation, 60% and 44% of patients were already extubated and classified as PGD_0_ in the non-ECMO and ECMO-groups, respectively. PGD grading was similar in the two groups (Table 1). Median survival of the whole population was 33 months (Q1–Q3: 16–48). No difference in overall survival was detected between the ECMO and non-ECMO groups (Figure 2).

### Morphological and Molecular Findings

**Figure 3:**
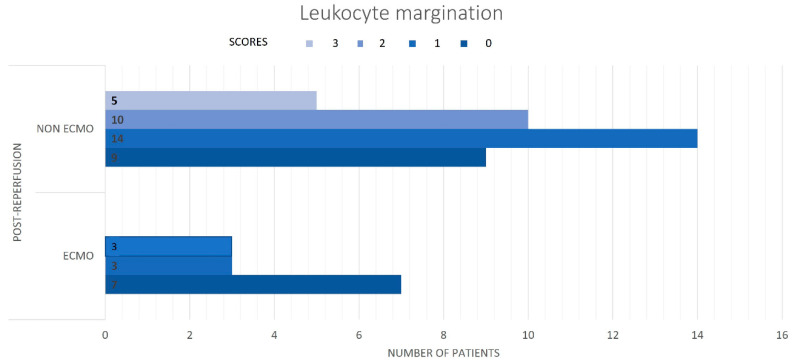
Distribution of scores for leukocyte margination in ECMO and non-ECMO patients post-reperfusion. In patients without ECMO, leukocyte margination scores were mostly 1–3 rather than 0.

**Figure 4:**
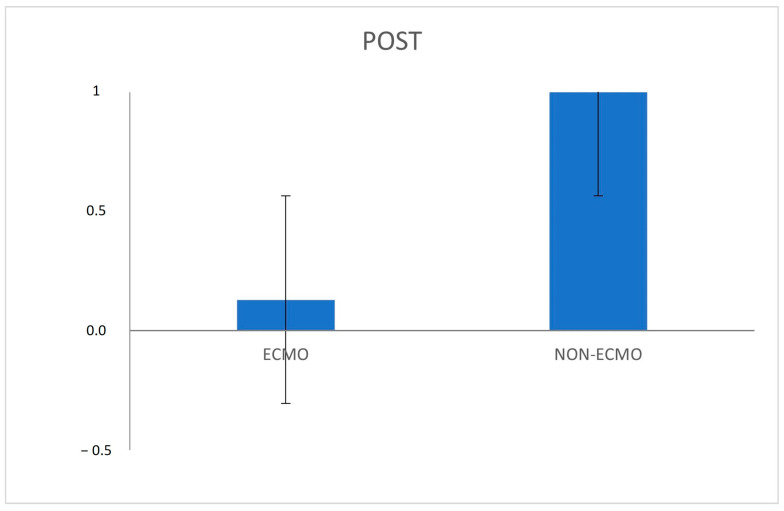
Real-time PCR for iNOS expression performed in 12 cases (6 ECMO and 6 non-ECMO patients post-reperfusion). Fold changes between ECMO and non-ECMO patients post-reperfusion showed higher median ΔCt values in the non ECMO group in comparison to the ECMO group, even if not significant. Data are expressed as 2-°∆Ct values (the value was assumed to be 1 for the ECMO group).

**Figure 5:**
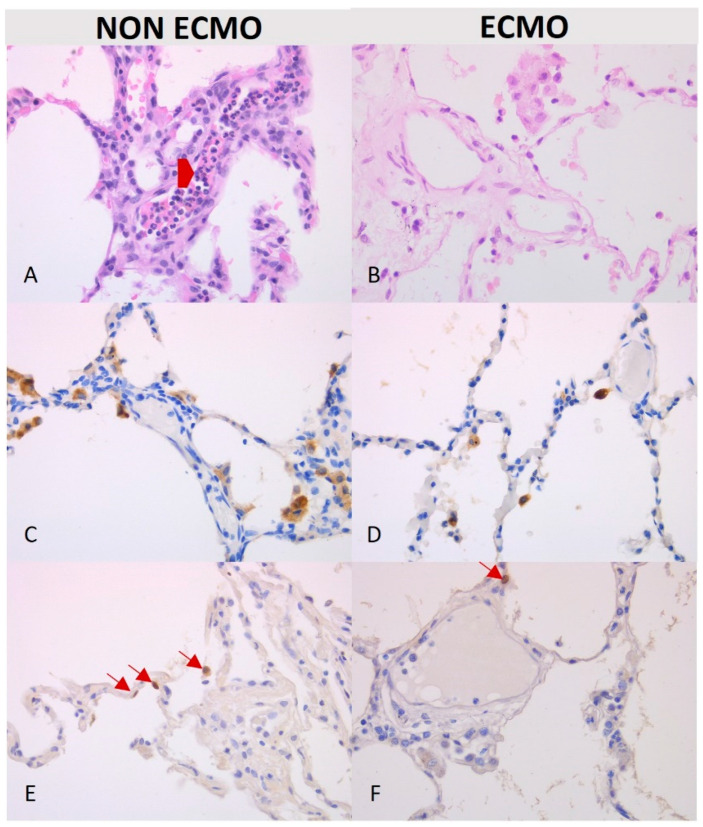
Explanatory figures for the ECMO and non-ECMO patients. Leukocyte margination ((**A**,**B**) hematoxylin and eosin staining, 40× original magnification, red arrowhead), iNOS expression ((**C**,**D**) immunohistochemistry, 40× original magnification), and apoptotic cells (dark nuclei, red arrows) ((**E**,**F**) tunel assay, 40× original magnification) are more evident in non-ECMO than in ECMO patients.

The histological examination of post-reperfusion biopsies in ECMO patients did not detect microthrombosis in any of the sections. In the pre-reperfusion time, samples from the ECMO and non-ECMO groups showed similar histological, immunohistochemical, and molecular features (Table 3). Interestingly, samples from ECMO patients showed several pathological differences in post-reperfusion biopsies compared to those from non-ECMO tissues. Post-reperfusion samples from the ECMO group showed statistically significantly lower leukocyte margination (*p* = 0.036) (Table 3, Figure 3) and lower congestion/blood extravasation scores (even if not significant) (Table 3) than non-ECMO samples. iNOS values were lower in the ECMO group in comparison to the non-ECMO group, even if not significant, as also demonstrated by the results of PCR (Figure 4). Explanatory figures of the histological findings for leukocyte margination, iNOS expression, and apoptotic cells in ECMO and non-ECMO patients are presented in Figure 5.

**Figure 6:**
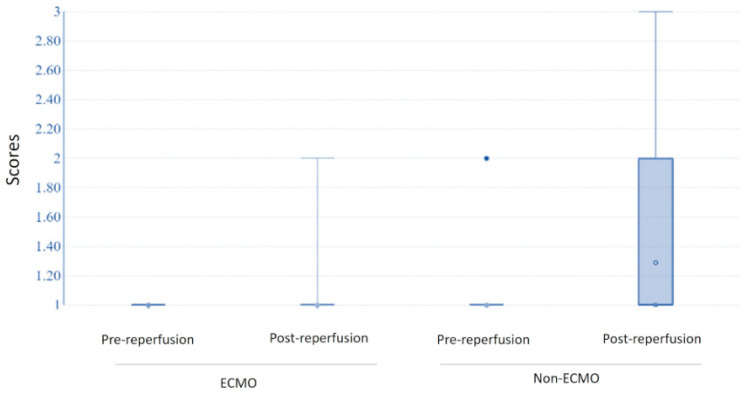
Leukocyte margination increasing between pre- and post-reperfusion biopsies in the ECMO and non-ECMO groups.

**Figure 7:**
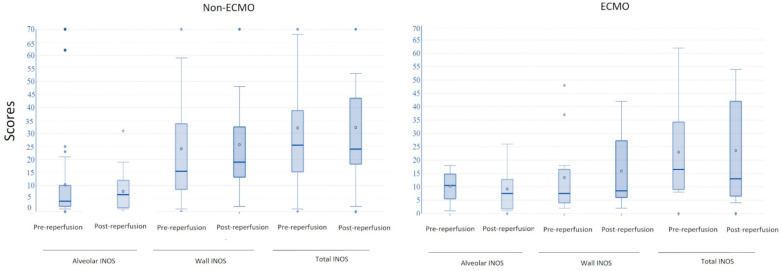
Comparison of pre- and post- reperfusion biopsies for alveolar, wall, and total INOS in non-ECMO and ECMO patients showing the tendency of iNOS values to increase in post-reperfusion biopsies of non-ECMO patients.

From the comparison of the interquartile ranges for the ECMO and non-ECMO groups, biopsies from non-ECMO patients showed a high grade of variability with a strong tendency for the scores of most parameters to increase between the pre- and post-reperfusion biopsies (Table 3, Table 4 and Table 5, Figure 6 and Figure 7).

The results for the comparison of the variability in the interquartile ranges of apoptotic indexes and INOS values are summarized in Table 4.

For each parameter, two models were estimated, one for each time-point (pre- and post-reperfusion), for ECMO vs. non-ECMO patients. The variables edema and congestion were dichotomized due to the poor representation of the categories (0: scores 0–1 and 1: scores 2–3) (Table 5). 

The combined score including morphological parameters, AI, and alveolar iNOS showed a trend of association with PGD at 24 and 48 h, even though not significant (*p* = 0.09 and *p* = 0.07, respectively). When considering morphological parameters and AI, the association with PGD was statistically significant at 72 h (*p* = 0.04).

## 4. Discussion

The present study reports our institutional experience with the use of intraoperative ECMO support during transplantation. The study demonstrated for the first time that lung tissues from ECMO were comparable, or for some parameters better, than non-ECMO.

Today, ECMO—VA-ECMO, in particular—is the technique most frequently used during lung transplantation [6]. When VA-ECMO is used, the first lung implanted is less exposed to high ventilation pressure because of the employment of lung-protective ventilation strategies, thus reducing alveolar overextension [6]. Moreover, the pulmonary blood flow is also reduced, thus better controlling reperfusion on the damaged vascular bed [4,19,20,21,22]. Currently, transplantation with or without ECMO support has been compared in only a few studies, showing better PGD, in particular at 72 h, and increased overall survival in patients who undergo ECMO [11,13].

Interestingly, an important result of our study is precisely the correlation between the severity of PGD at 72 h and the combined injury scores for post-reperfusion biopsies. Should our results be confirmed, this study may provide the basis for understanding pathological substrates that cooperate in the development of the clinical behavior.

Indeed, no other works have explored the pathological roots of the beneficial effect of ECMO in lung transplantation. Leukocyte margination was found to be statistically significantly lower in the ECMO than in the non-ECMO group, which may be directly correlated with the lower stress to which the vascular compartment is subjected. This may result in a reduction of intrinsic inflammation in this site, with a few chemoattractive stimuli and insignificant expression of adhesion proteins on the endothelial cell surface, thus not recalling leukocytes [23].

Currently, there is an unmet need to find a histological injury scoring system to quantify the morphological changes (edema, blood extravasation, and leukocyte margination) that usually occur during the preservation and reperfusion of transplanted lungs.

Experimental studies have shown that long periods of cold ischemia (>12 h) before reperfusion are associated with more extensive tissue-injury-detecting cell death, primarily of necrotic type [24]. Necrosis was never detected in our samples, as the maximum ischemia time was 10 h; however, we cannot rule out that microscopic necrotic areas could have been present but were not detected in our biopsies. Several clinical and experimental studies have demonstrated that apoptosis is the principal mode of cell death occurring after I/R injury [18,25]. In the present study, we detected very low AI in both groups, even if alveolar iNOS expression was lower in the lung samples from the ECMO group. During I/R time, high concentrations of NO are produced due to the activation of several types of NO synthases, but the role of this molecule is highly debated in the literature [26]. Some authors have suggested that NO can be either protective or toxic to lung grafts depending on dose, timing, and duration of exposure and that NO has a narrow therapeutic window [27]. It is now widely accepted by several authors that NO has a detrimental effect after lung transplantation when released at high concentrations: combined with reactive oxygen species, it produces peroxynitrite and other reactive nitrogen species, resulting in severe cellular damage [27]. Two different principal NO synthases are involved in regulating pulmonary vascular function: endothelial NOS (eNOS) and iNOS, principally expressed by inflammatory cells [27]. Several experimental studies have demonstrated that inhibition of iNOS with iNOS inhibitors or siRNA lowers hypoxia-induced increases in NO production, lipid peroxidation, LTB4 levels, caspase-3 activity, and apoptosis [27].

In our study, the combined injury score system, which included several parameters (edema, congestion/blood extravasation, leukocyte margination, AI, and alveolar iNOS), showed a better performance in reflecting PGD status. This had two important results: (i) it showed the critical impact of apoptotic cell death and iNOS in I/R injury; and (ii) it suggested that the association of multiple parameters may be more representative of the clinical setting by weighing the contribution of various factors in the context of a complex mechanism, such as PGD. Large case series and mechanistic studies are needed to confirm the key role of iNOS in inducing I/R apoptotic cell death.

Another interesting result of our study was the variability in the rates for all morphological parameters in the ECMO group, which were significantly lower than those in the non-ECMO group. A plausible explanation of reduced variability in all morphological parameters may be related to the aforementioned protective action of ECMO on pulmonary blood flow and alveolar overextension, resulting in greater control of hemodynamic and ventilator conditions, with consequently more stable parameters during surgical procedures.

Our study has several limitations. First, it is a single-center retrospective analysis, the sample size is relatively small, and some clinical information is missing. However, the statistical techniques used in the study were among those best suited for dealing with a limited number of patients and a large number of covariates. The case series was recruited over a relatively short time, thus guaranteeing a certain uniformity in treatment modalities. In our investigation, patients belonging to the ECMO group were usually more complex, and this may have represented a selection bias. However, this assumption could further strengthen our results. Indeed, as patients transplanted into ECMO had higher pulmonary pressures, they should have had more severe IR injury and thus more severe PGD [12,28]^.^ Another important limitation of the study, which, however, can further strengthen the results, is represented by the fact that within the ECMO group there were patients in whom the ECMO support was pre-emptive and patients in whom the support was of necessity due to intraoperative cardio-respiratory instability. Obviously, this assumes that the first type of patients benefited most from the positive effects of ECMO support.

Moreover, following ISHLT recommendations [17], recipients with post-operative ECMO should have grade 3 PGD by definition. However, this classification does not consider the recent strategy in the use of ECMO for prophylactic purposes in patients at high risk of PGD post-surgery, as introduced by the Vienna group [12,13] and also applied in our clinical practice. In our series, there were only three patients with prolonged ECMO, and therefore we could not extrapolate statistically significant data. These patients were classified as PGD grade 3, even if they had a clear chest X-ray.

For these reasons, in our study, a clear reduction in the incidence of clinical PGD was not evident, although a positive trend in patients supported by ECMO was detected.

## 5. Conclusions

These preliminary data suggest low-grade tissue damage (as determined by low leukocyte margination and combined scores for ischemia/reperfusion injury) in patients supported by intraoperative ECMO. The use of larger, multicentric, and prospective case series, with complete clinical data and more homogeneous treatments (e.g., all patients treated with pre-emptive central VA-ECMO), is mandatory to validate and confirm our findings in order to determine the role of intraoperative ECMO in the clinical management of lung transplantation.

## Figures and Tables

**Table 1:** Study population: descriptive statistics. Continuous data are reported as medians (I, III quartiles); categorical data are reported as percentages and absolute frequencies. Wilcoxon-type tests were performed for continuous variables, and Pearson chi-square tests were performed for categorical variables.

	*n*	Non ECMO (*n* = 38)	ECMO (*n* = 13)	Combined (*n* = 51)	*p*-Value
Age	51	36.00/49.00/57.75	45.00/56.00/60.00	36.00/52.00/58.00	0.264
Gender: M	51	37% (14)	23% (3)	33% (17)	0.363
F		63% (24)	77% (10)	67% (34)	
Native disease: cystic fibrosis	51	11% (4)	8% (1)	10% (5)	0.577
COPD		47% (18)	54% (7)	49% (25)	
ILD		37% (14)	23% (3)	33% (17)	
Other		5% (2)	15% (2)	8% (4)	
Weight	51	50.5/64.0/77.5	65.0/78.0/86.0	54.5/65.0/81.5	0.058
Height	51	160.0/170.0/177.0	165.0/169.0/176.0	160.5/170.0/177.0	0.881
BMI	51	19.50781/21.77679/25.11628	21.97134/26.19619/29.93827	19.97871/23.09541/27.65886	0.016 *
Pulmonary arterial pression (mmHg)	49	17.00/22.00/25.25	25.00/31.00/35.00	18.00/22.00/27.00	0.004 *
Total lung ischemia time (min)	45	219.50/270.00/317.50	206.25/251.00/299.75	215.00/267.00/315.00	0.388
Primary graft dysfunction h 0: 0–1	30	24 62% (15)	6 20% (1)	53% (16)	0.08
2–3		38% (9)	80% (5)	47% (14)	
Primary graft dysfunction h 24: 0–1	32	25 70% (17)	7 30% (2)	59% (19)	0.09
2–3		30% (8)	70% (5)	41% (13)	
Primary graft dysfunction h 48: 0–1	30	25 70% (18)	5 40% (2)	67% (20)	0.3
2–3		30% (7)	60% (3)	33% (10)	
Primary graft dysfunction h 72: 0–1	33	25 80% (19)	8 50% (4)	70% (23)	0.2
2–3		20% (6)	50% (4)	30% (10)	
Early mortality (<30 days)	51	5.3% (2)	0	3.9% (2)	-

* for statistical significance. Abbreviations: M: male, F: female; BMI: body mass index; COPD: chronic obstructive pulmonary disease; ILD: interstitial lung disease.

**Table 2:** Donor characteristics: descriptive statistics. Continuous data are reported as medians (I, III quartiles); categorical data are reported as percentages and absolute frequencies. Wilcoxon-type tests were performed for continuous variables, and Pearson chi-square tests were performed for categorical variables.

	*n*	Non-ECMO (*n* = 38)	ECMO (*n* = 13)	Combined (*n* = 51)	*p*-Value
Age	51	28.00/43.00/52.75	33.00/47.00/54.00	29.50/44.00/53.00	0.461
Gender: M	51	55% (21)	77% (10)	61% (31)	0.167
F		45% (17)	23% (3)	39% (20)	
Cause of death: Trauma	48	34% (12)	38% (5)	35% (17)	0.824
Cardiovascular events		51% (18)	54% (7)	52% (25)	
Other		14% (5)	8% (1)	12% (6)	
Alcohol: No	50	95% (36)	83% (10)	92% (46)	0.204
Yes		5% (2)	17% (2)	8% (4)	
Smoking: No	50	82% (31)	58% (7)	76% (38)	0.1
Yes		18% (7)	42% (5)	24% (12)	
Oto score	51	1/2/4	1/3/4	1/3/4	0.779

Abbreviations: M: male, F: female.

**Table 3:** Donor histological findings: descriptive statistics. Continuous data are reported as medians (I, III quartiles); categorical data are reported as percentages and absolute frequencies. Wilcoxon-type tests were performed for continuous variables, and Pearson chi-square tests were performed for categorical variables.

	Pre-Reperfusion			Post-Reperfusion		
Variables	ECMO Group (*n* = 13)	Non-ECMO Group (*n* = 38)	*p*-Value	ECMO Group (*n* = 13)	Non-ECMO Group (*n* = 38)	*p*-Value
Edema (median; IQR)	0 (0–0)	0 (0–0)	0.30 0.57	0 (0–0)	0 (0–0)	0.86 0.62
0 (*n*, %)	13 (100%)	37 (97%)		12 (92%)	33 (87%)	
1	0	1 (3%)		0	4 (10%)	
2	0	0		0	1 (3%)	
3	0	0		1 (8%)	0	
Congestion/blood extravasation (median; IQR)	0 (0–0)	1 (0–1)	0.39 0.42	0 (0.75–2)	1 (0–2)	0.95 0.98
0 (*n*, %)	10 (77%)	25 (66%)		3 (23%)	13 (34%)	
1	2 (15%)	10 (26%)		6 (46%)	10 (26%)	
2	1 (8%)	3 (8%)		3 (23%)	11 (29%)	
3	0	0		1 (8%)	4 (11%)	
Leucocyte margination (median; IQR)	0 (0–0)	0 (0–0)	0.5 0.29	0 (0–1)	1 (1–2)	0.036 * 0.03 *
0 (*n*, %)	11 (85%)	30 (79%)		7 (70%)	9 (24%)	
1	2 (15%)	6 (16%)		1 (10%)	14 (37%)	
2	0	2 (5%)		2 (20%)	10 (26%)	
3	0	0		0	5 (13%)	
Apoptotic index (median; IQR)	0.67 (0–3.55)	0.67 (0.33–1.33)	0.89	0 (0–0.415)	0.415 (0–0.67)	0.48
iNOS (median; IQR)						
Alveolar	10.5 (5.5–14.75)	4.15 (2–19)	0.07	7.5 (1.75–12.75)	6.6 (1.5–12)	0.71
Wall	7.5 (4–16.5)	15.5 (8.55–33.75)	0.12	8.5 (8–27.25)	19 (13.25–32.5)	0.2
Total	16.5 (9–34.25)	25.5 (15.5–38.75)	0.68	13 (6.5–42)	24 (18.25–43.5)	0.6

* for statistical significance. Abbreviations: IQR: interquartile range.

**Table 4:** Comparison of the variability in the interquartile ranges of apoptotic indexes and INOS values. A *p*-value < 0.05 indicates a statistically significant difference in variability (measured as an interquartile range).

Group of Comparison	Parameter	*p*-Boot
Pre- and post-reperfusion in ECMO	Apoptotic index	0.308
	Intra-alveolar INOS	0.0345 *
	Wall INOS	0.0064 *
	Total INOS	0.0061 *
Pre- and post-reperfusion in non-ECMO	Apoptotic index	0.790
	Intra-alveolar INOS	0.1361
	Wall INOS	0.0501 *
	Total INOS	0.0424 *
ECMO vs. non-ECMO	Apoptotic index	0.143
	Intra-alveolar INOS	0.0484 *
	Wall INOS	0.0154 *
	Total INOS	0.0069 *

* for statistical significance.

**Table 5:** Regression models at two time-points for each parameter in ECMO vs. non-ECMO patients.

Parameter	Time Points	Coefficient	CI.95	*p*-Value
Edema	Pre-reperfusion	Not estimable	-	-
	Post-reperfusion	0.44	[0.05;4.09]	0.4737
Congestion	Pre-reperfusion	0.58	[0.13;2.47]	0.4584
	Post-reperfusion	1.73	[0.41;7.42]	0.4584
Leukocyte margination	Pre-reperfusion	−0.11	[−0.43;0.22]	0.513006
	Post-reperfusion	−0.67	[−1.26;−0.08]	0.0295 *
Apoptotic index	Pre-reperfusion	0.79	[−0.47;2.05]	0.2258226
	Post-reperfusion	−0.51	[−1.51;0.49]	0.322918
Alveolar iNOS	Pre-reperfusion	−0.08	[−9.61;9.44]	0.9865585
	Post-reperfusion	7.77	[5.21;10.34]	<1 × 10^−4^ *
Wall iNOS	Pre-reperfusion	−10.63	[−24.00;2.74]	0.1264
	Post-reperfusion	−9.74	[−23.06;3.58]	0.1587
Total iNOS	Pre-reperfusion	−9.14	[−25.10;6.83]	0.268
	Post-reperfusion	−8.70	[−24.13;6.73]	0.2751

* for statistical significance.

## Data Availability

All relevant data were reported within the article. Further supporting data will be provided upon written request addressed to the corresponding author.
